# Hydroxymethylation profile of cell-free DNA is a biomarker for early colorectal cancer

**DOI:** 10.1038/s41598-022-20975-1

**Published:** 2022-10-04

**Authors:** Nicolas J. Walker, Mamunur Rashid, Shirong Yu, Helen Bignell, Casper K. Lumby, Carmen M. Livi, Kate Howell, David J. Morley, Sandro Morganella, Daniel Barrell, Shabhonam Caim, Walraj Gosal, Jens Füllgrabe, Thomas J. Charlesworth, Louella Vasquez, Miika Ahdesmäki, Jordan Eizenga, Parul Prabhat, Vitali Proutski, Marie Laurie Murat-Onana, Catherine J. Greenwood, Lisa Kirkwood, Meeta Maisuria-Armer, Mengjie Li, Emma Coats, Victoria Winfield, Lachlan MacBean, Toby Stock, Alice Tomé-Fernandez, Yat Chan, Nasir Sheikh, Paula Golder, Michael Steward, Tobias W. B. Ost, Douglas Stewart, Albert Vilella, Mojtaba Noursalehi, Benedict Paten, Debora Lucarelli, Joanne Mason, Gareth Ridge, Jason Mellad, Suman Shirodkar, Shankar Balasubaramanian, Joanna D. Holbrook

**Affiliations:** 1 Cambridge Epigenetix, Saffron Walden, UK; 2 Amanita Informatics, LLC, 100 Quarry Ln, Santa Cruz, CA USA; 3 UC Santa Cruz Genomics Institute, University of California, Santa Cruz, CA USA; 4 Biostatistics and Research, LLC, Cambridge, USA; 5 Department of Chemistry, University of Cambridge, Cambridge, UK; 6 Cancer Research UK, Cambridge Institute, University of Cambridge, Cambridge, UK

**Keywords:** Cancer genomics, DNA methylation, Epigenomics

## Abstract

Early detection of cancer will improve survival rates. The blood biomarker 5-hydroxymethylcytosine has been shown to discriminate cancer. In a large covariate-controlled study of over two thousand individual blood samples, we created, tested and explored the properties of a 5-hydroxymethylcytosine-based classifier to detect colorectal cancer (CRC). In an independent validation sample set, the classifier discriminated CRC samples from controls with an area under the receiver operating characteristic curve (AUC) of 90% (95% CI [87, 93]). Sensitivity was 55% at 95% specificity. Performance was similar for early stage 1 (AUC 89%; 95% CI [83, 94]) and late stage 4 CRC (AUC 94%; 95% CI [89, 98]). The classifier could detect CRC even when the proportion of tumor DNA in blood was undetectable by other methods. Expanding the classifier to include information about cell-free DNA fragment size and abundance across the genome led to gains in sensitivity (63% at 95% specificity), with similar overall performance (AUC 91%; 95% CI [89, 94]). We confirm that 5-hydroxymethylcytosine can be used to detect CRC, even in early-stage disease. Therefore, the inclusion of 5-hydroxymethylcytosine in multianalyte testing could improve sensitivity for the detection of early-stage cancer.

## Introduction

The detection and treatment of cancer when the disease is still at an early stage could save many lives, reduce morbidity, and relieve the burden of cancer on healthcare systems^1,2^.

Liquid biopsy is a minimally invasive approach to detect early cancer in body fluids such as blood and urine^3–5^. There are many challenges to developing a liquid biopsy test that is sufficiently powerful to detect cancer, especially in early-stage disease when the tumor is releasing only minute amounts of biomarkers into the bloodstream. Circulating tumor DNA (ctDNA) represents just 0.1–1% of overall cell-free DNA (cfDNA) in early disease^6–8^. Multianalyte approaches that measure multiple biomarkers from the same blood sample could be useful for the detection of early-stage cancer^9,10^.

Changes in the epigenome may precede genetic changes in tumorigenesis^11^. Therefore, there is growing interest in utilizing the epigenome of cfDNA for cancer detection. Several groups have investigated whether cancer can be detected via epigenetic modifications in the tumor fraction of cfDNA, due to DNA methylation^8,12^, DNA hydroxymethylation^13–15^ and cfDNA characteristics which may reveal chromatin structure^16^.

Methylation of cystosine bases to produce methylcytosine (5mC) is a well-known epigenetic mechanism controlling gene expression. 5mC is oxidized to form 5-hydroxymethylcytosine (5hmC). 5mC and 5hmC have different functional roles: 5mC is present in heterochromatin and euchromatin and generally represses gene expression^17^, whereas 5hmC is mainly present in euchromatin and is associated with the mostly highly transcribed gene bodies and their enhancers^18,19^, as well as with poised enhancers^20^. 5mC and 5hmC have differential affinity to epigenetic readers; for instance, methyl binding proteins (MBD) preferentially bind 5mC. UHRF2 has been reported to have preferential affinity for 5hmC^21^, and there are a limited number of proteins that bind both modifications, such as MeCP2^22–24^. Both 5mC and 5hmC are actively replaced after mitosis^25^.

The different functional roles and distribution in cancer samples suggest that 5mC and 5hmC have independent utility as biomarkers^22,26^. However, to date, the hunt for epigenetic markers of cancer has been constrained by available technologies, with limited options available to distinguish between 5mC and 5hmC. Efforts have traditionally focused on the use of bisulfite to sequence both 5mC and 5hmC without distinguishing one from another^8,27^.

Recently however, several techniques have emerged to quantify and utilize 5hmC signatures for cancer detection via liquid biopsy^13–15,28,29^. The information provided about cancer by 5hmC profiles has been shown to be orthogonal and additive to 5mC^14,30^. However, studies in colorectal cancer (CRC) have been somewhat limited by their small sample size and limited quantitative performance of the methodology used^13–15,28,29^. In addition, the observation that 5hmC is progressively lost in later, metastatic cancers^13,31^ suggests that its power as a biomarker may be stage specific. We set out to further quantify and validate 5hmC’s potential in a robust study of CRC, with collection and incorporation of covariate information and a sample set sufficient to study stage-specific performance.

In this study, we measured 5hmC levels across the cell-free genome and trained a classifier to distinguish individuals with colorectal cancer (CRC) from controls. We assessed the performance of the classifier in an independent validation set and assessed its dependence on cancer stage and ctDNA levels. We evaluated how classifier performance evolved when orthogonal information about cfDNA fragment characteristics was added to 5hmC information. The study showed that 5hmC profiles of cfDNA are a strong predictor of cancer, including high sensitivity for the detection of early-stage cancer. The performance achieved in this large well-controlled study with internal validation is at least comparable to performance in CRC reported for other analytes^8,12,32–34^ and for 5hmC in smaller studies^13,14^. Our data suggest that the 5hmC signal derived from epigenetic changes in cfDNA is more sensitive at an early stage than that from other analytes. This sensitivity is retained when the 5hmC signal is combined with cfDNA fragment characteristics to produce an additive signal.

## Results

### Study population

**Figure 1:**
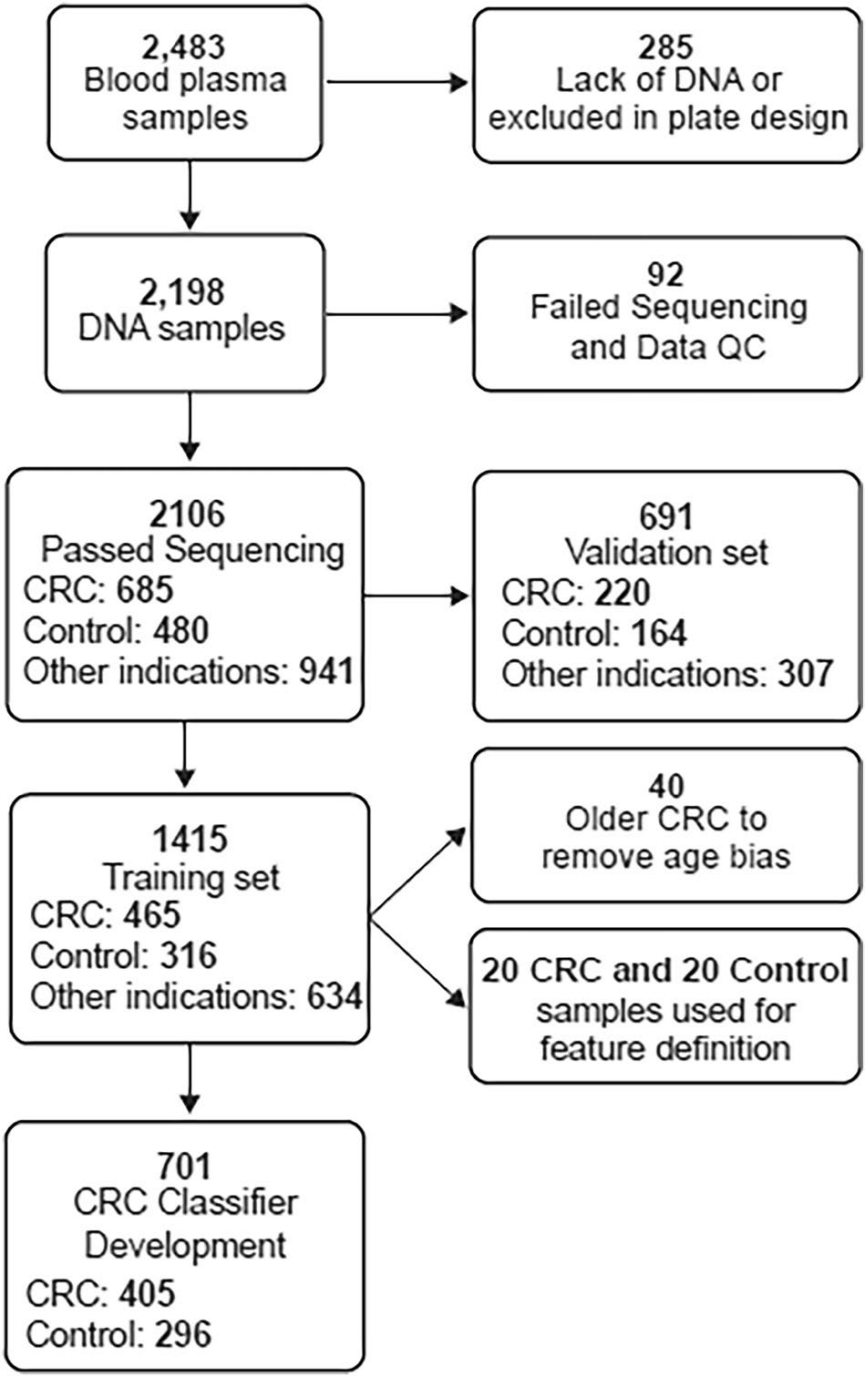
Flow chart of subjects included in the study. Control samples were made up of individuals who were CRC and adenomatous polyp negative (colonoscopy confirmed). A total of 8.3% of the control individuals were diagnosed with peptic ulcers, arthritis or COPD.

Blood samples were donated by 2483 individuals prior to undergoing colonoscopy. These double-spun plasma samples were purchased from multiple vendors and included biobanked and prospectively collected samples. Experimental batches for cfDNA extraction, hydroxymethylome library preparation and sequencing were balanced for key sample characteristics (vendor, age, sex, ethnicity and diagnosis) (Fig. 1, Tables 1, 2). The cfDNA from each individual was processed to generate two sequencing libraries: a whole genome library (denoted “input”) and a hydroxymethylome library.

**Table 1:** Demographics of the whole cohort (n = 2483), training set (n = 781 control and CRC samples) and validation set (n = 384 control and CRC samples).

Participating individuals						
Study	Total	Control*	Other cancers**	CRC		
N	2483	573	1113	797		
Age (years), mean (SD)	64.19(9.11)	62.24(8.99)	63.97(8.79)	65.70(9.37)		
Female gender, n (%)	1283 (51.67%)	318 (55.50%)	559 (50.22%)	406 (50.94%)		
**Ethnicity, n (%)**						
Asian	270 (10.87%)	61 (10.65%)	122 (10.96%)	87 (10.92%)		
Black/African American	52 (2.09%)	12 (2.09%)	39 (3.50%)	1 (0.13%)		
Pacific Islander	55 (2.22%)	0 (0.00%)	1 (0.09%)	54 (6.78%)		
Other	274 (11.04%)	104 (18.15%)	62 (5.57%)	108 (13.55%)		
Unknown	23 (0.93%)	0 (0.00%)	16 (1.44%)	7 (0.88%)		
White	1809 (72.86%)	396 (69.11%)	873 (78.44%)	540 (67.75%)		

	Control*	CRC	CRC stage 1	CRC stage 2	CRC stage 3	CRC stage 4
**Training set**						
N	316	465	91	189	135	50
Age, years, mean (SD)	61.74 (8.89)	65.51 (9.13	66.20 (8.20)	65.78 (9.31)	65.19 (9.78)	64.06 (8.22)
Female gender, n (%)	174 (55.06%)	244 (52.47%)	50 (54.95%)	98 (51.85%)	70 (51.85%)	26 (52.00%)
Ethnicity, n (%)						
Asian	15 (4.75%)	51 (10.97%)	16 (17.58%)	13 (6.88%)	18 (13.33%)	4 (8.00%)
Black/African American	10 (3.16%)	0 (0.00%)	0 (0.00%)	0 (0.00%)	0 (0.00%)	0 (0.00%)
Pacific Islander	0 (0.00%)	26 (5.59%)	3 (3.30%)	23 (12.17%)	0 (0.00%)	0 (0.00%)
Other	71 (22.47%)	73 (15.70%)	2 (2.20%)	28 (14.81%)	30 (22.22%)	13 (26.00%)
Unknown	0 (0.00%)	5 (1.08%)	0 (0.00%)	5 (2.65%)	0 (0.00%)	0 (0.00%)
White	220 (69.62%)	310 (66.67%)	70 (76.92%)	120 (63.49%)	87 (64.44%)	33 (66.00%)
**Validation set**						
N	164	220	45	87	61	27
Age (years), mean (SD)	65.88 (9.64)	62.46 (9.24)	64.31 (7.54)	66.41 (10.05)	65.49 (10.29)	67.63 (10.00)
Female gender, n (%)	95 (57.93%)	116 (52.73%)	21 (46.67%)	49 (56.32%)	35 (57.38%)	11 (40.74%)
Ethnicity, n (%)						
Asian	10 (6.10%)	24 (10.91%)	5 (11.11%)	8 (9.20%)	6 (9.84%)	5 (18.52%)
Black/African American	1 (0.61%)	1 (0.45%)	0 (0.00%)	1 (1.15%)	0 (0.00%)	0 (0.00%)
Pacific Islander	0 (0.00%)	15 (6.82%)	2 (4.44%)	13 (14.94%)	0 (0.00%)	0 (0.00%)
Other	30 (18.29%)	24 (10.91%)	0 (0.00%)	5 (5.75%)	14 (22.95%)	5 (18.52%)
Unknown	0 (0.00%)	1 (0.45%)	0 (0.00%)	1 (1.15%)	0 (0.00%)	0 (0.00%)
White	123 (75.00%)	155 (70.45%)	38 (84.44%)	59 (67.82%)	41 (67.21%)	17 (62.96%)

*Control samples were from individuals with conditions including rheumatoid arthritis, COPD and peptic ulcer.

**Other cancer samples were from individuals with the following cancers: advanced adenoma, breast cancer, lung cancer, non-advanced adenoma, ovarian cancer, prostate cancer, stomach cancer, urinary cancer.

**Table 2:** Disease characteristics of whole cohort.

N	Total cohort		Validation set		Training set	
	2483	%	691	%	1415	%
Colorectal cancer	797	32.10	220	31.84	465	32.86
CRC-1	161	6.48	45	6.51	91	6.43
CRC-2	319	12.85	87	12.59	189	13.36
CRC-3	222	8.94	61	8.83	135	9.54
CRC-4	95	3.83	27	3.91	50	3.53
**Other cancers**						
Advanced adenoma	306	12.32	82	11.87	169	11.94
Breast cancer	114	4.59	34	4.92	68	4.81
Lung cancer	140	5.64	51	7.38	80	5.65
Non-advanced adenoma	373	15.02	88	12.74	218	15.41
Ovarian cancer	32	1.29	10	1.45	15	1.06
Prostate cancer	91	3.66	25	3.62	50	3.53
Stomach cancer	32	1.29	8	1.16	21	1.48
Bladder cancer	25	1.01	9	1.30	13	0.92
**Control**						
Standard	531	21.39	149	21.56	291	20.57
Rheumatoid arthritis	20	0.81	7	1.01	11	0.78
Peptic ulcer	12	0.48	6	0.87	6	0.42
COPD	10	0.40	2	0.29	8	0.57

Sequenced libraries were assessed for quality by scoring samples across multiple parameters, including overall read count, spike-in control amplification and uniformity. Libraries for 2106 individuals (685 with CRC, 480 controls and 941 with other conditions such as adenoma and other cancers) successfully passed sequencing and quality control (Fig. 1). Of the 2106 individuals, mean age was 64 years, and 52% were female. Samples that did not produce successful libraries were excluded; in most cases this was due to poor cfDNA yield (Fig. 1) The individuals who donated these samples had similar characteristics to the 2106 individuals who comprised the study population (Tables 1, 2).

### 5hmC is efficiently captured from cfDNA to produce hydroxymethylome libraries

**Figure 2:**
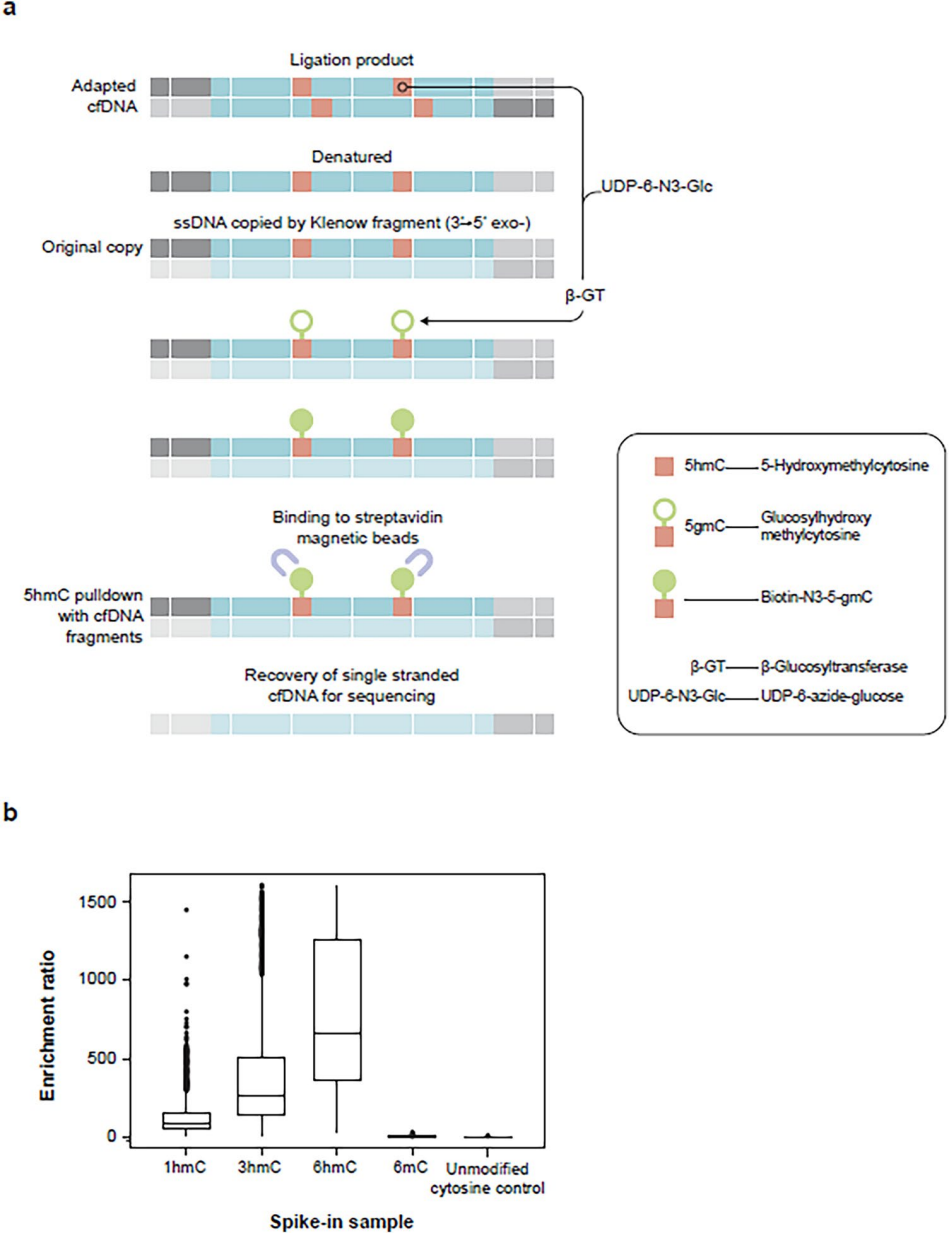
(**A**) Hydroxymethylome capture procedure. (**B**) 166 bp synthetic spike-in controls with 1, 3, 6 5hmC residues demonstrate that the hydroxymethylome enriches for 5hmC over controls containing 6 5mC residues and unmodified cytosines.

We captured the hydroxymethylome of cfDNA fragments containing 5hmC residues using just 5 ng of cfDNA. The high-throughput methodology has similarities to the technique described by Song et al.^13,18^ but differs in that DNA molecules labeled for 5hmC are copied and it is the copied strand that is captured, thus avoiding steric hindrance caused by the labels and ensuring quantitative capture of 5hmC density. The technique was automated on 96-well plates using liquid handlers. Briefly, cfDNA was extracted from 2 ml double spun plasma and quantified. Illumina-compatible sequencing libraries were prepared using 5 ng input cfDNA. A portion of this ‘input’ library was reserved for sequencing. The remaining sequencing library was denatured, and the single-stranded library was copied to create a double-stranded library where only one strand retained epigenetic information. 5hmC residues were enzymatically labeled with a modified glucose group, which was then biotinylated. 5hmC-containing double-stranded DNA fragments were captured using streptavidin beads. The copied strand without epigenetic modifications was recovered from the 5hmC-captured libraries and amplified to form the hydroxymethylome-enriched sequencing library (Fig. 2A).

Input and hydroxymethylome libraries were paired-end sequenced with an average of 62 M reads per library. The input libraries covered on average 85% of the human genome with at least one read and ~ 17% with a read depth of more than five reads. In contrast, the hydroxymethylome libraries were more localized to distinct genomic regions and tended to form peaks that could be characterized at both broad and narrow resolution and covered only 31% of the genome with at least one read and ~ 5% at more than five reads. This is consistent with previous reports describing the genomic distribution of 5hmC^13,15,35^.

5hmC was enriched in genic regions with an average ratio of 1.8 times that of intergenic regions, as many reads fell in the genic regions compared to intergenic regions. In contrast, input libraries had an average genic to intergenic ratio of 0.8. This is consistent with previous reports that cfDNA is preferentially hydroxymethylated in genic regions^14,15^. These metrics varied little between plates and processing batches (median absolute deviation was 0.02 for intra-plate technical replicates and 0.04 for inter-plate technical replicates).

Control DNA sequences were included in all samples to report the efficacy and quantitative nature of hydroxymethylome capture. The positive controls, containing 1, 3 or 6 5hmC residues, were enriched in the hydroxymethylome versus input libraries with 88-, 267- and 658-fold more reads, respectively. The negative controls, containing 6 mC residues or unmodified cytosines, were not enriched (1- and 0.38-fold, respectively) (Fig. 2B).

### 5hmC quantification and distribution

Machine learning feature sets were generated from the hydroxymethylome capture data by producing normalized ratios of read counts in the hydroxymethylome versus input sequencing libraries within coordinates of genes and enhancers.

**Figure 3:**
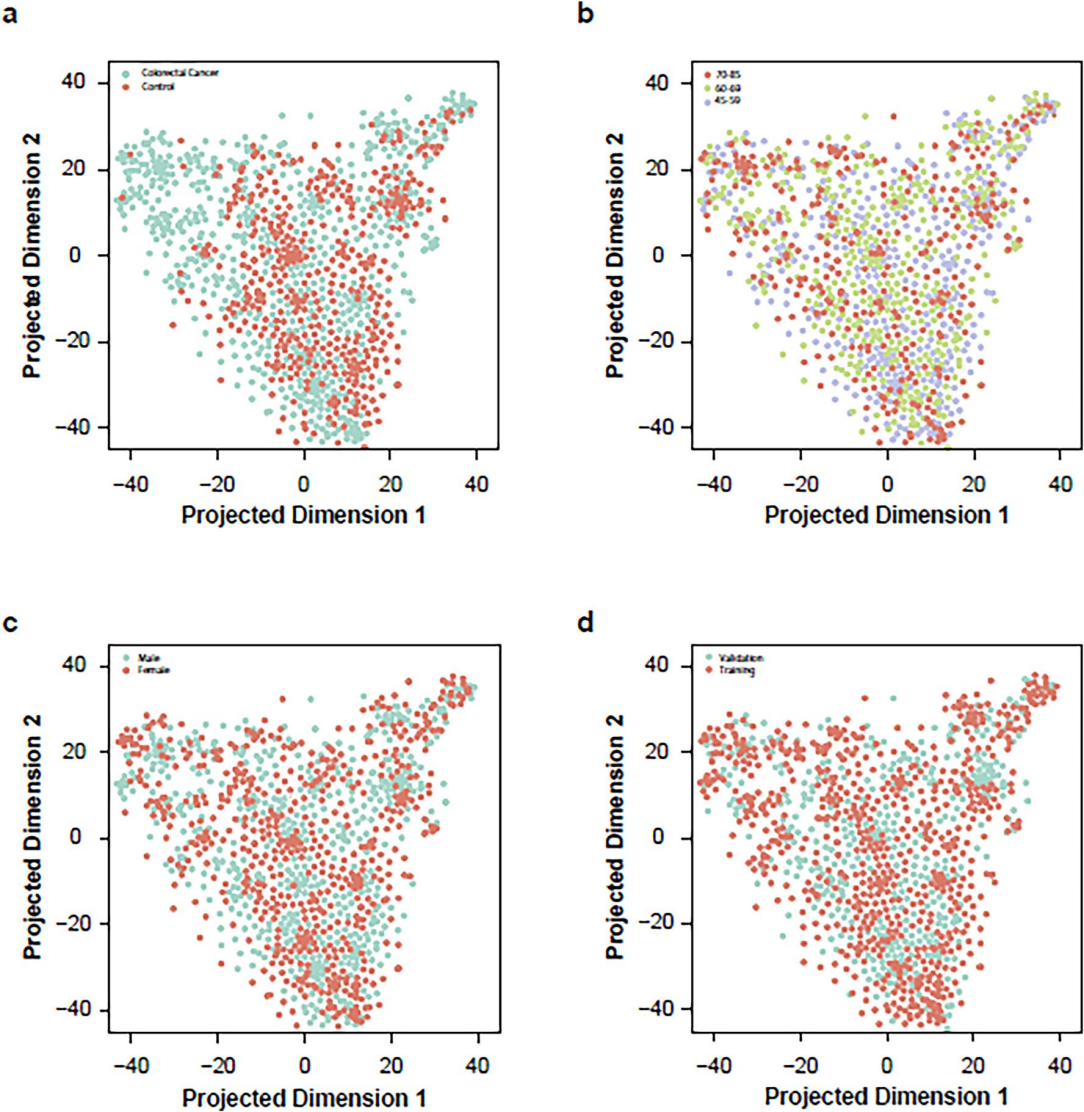
A two-dimensional representation of 5hmC quantified within gene enhancers over the training and validation samples displays evidence of clustering by disease status (CRC = green, control = orange), with little bias for gender (male = green, female = orange) or age (45–59, 60–69, 70–85 years) (t-SNE parameters: perplexity = 20, theta = 0.5).

An unsupervised t-distributed stochastic neighbor embedding (t-SNE) analysis showed some separation of CRC and control samples across projected dimension 1 but with substantial overlap between the classes (Fig. 3). No separation was observed for covariates such as gender and age (Fig. 3).

### 5hmC classifier detects CRC, even at early stage

5hmC levels in enhancer regions were used to train a supervised classifier algorithm with an ensemble of 50 learners to distinguish CRC from controls (405 CRC samples and 296 controls, Fig. 1). The area under the receiver operator curve (AUC) achieved in cross-validation within the training set was 90%, with 63% sensitivity at 95% specificity.

**Figure 4:**
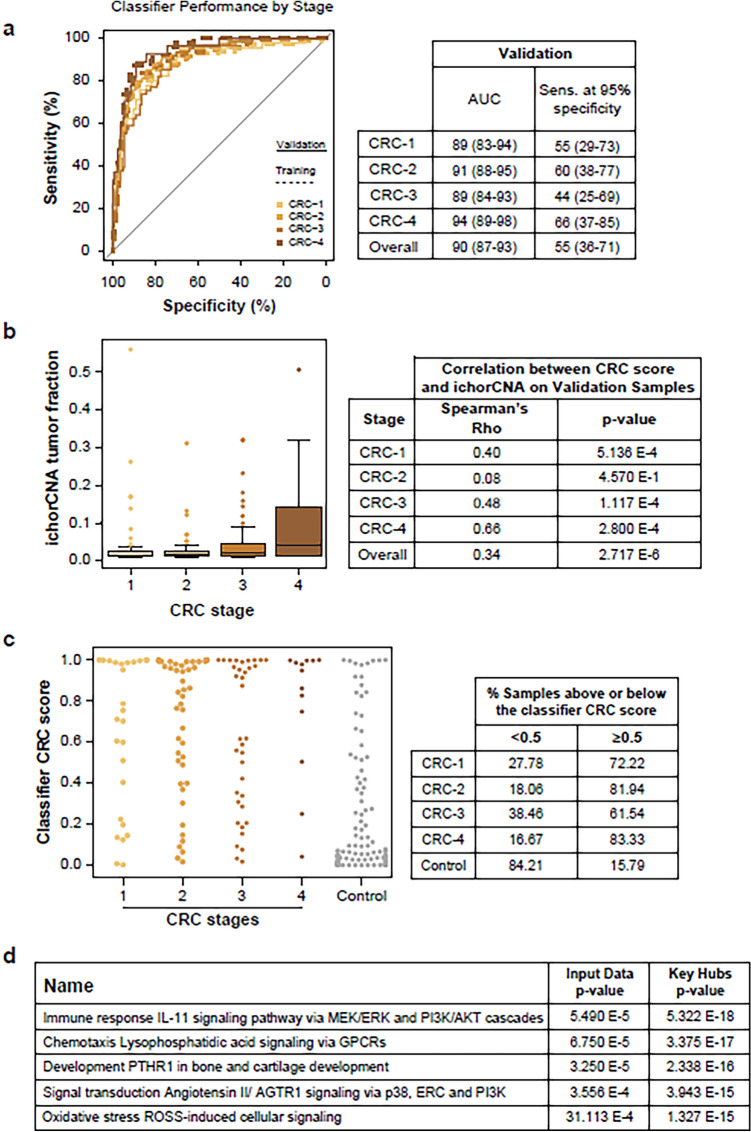
(**A**) Classifier trained on 5hmC levels in enhancer regions shows equivalent performance on the training (dotted line) and validation sets (solid line) and high performance across all CRC stages versus controls, with AUCs ranging from 88.6 to 93.6%. Cut-of-values are reported in Supp. Table 1. (**B**) The IchorCNA tumor fraction was positively correlated with tumor stage in validation samples. The correlation with the CRC classifier score was lower in early-stage samples (stages 1 and 2), with higher p-values than in later-stage samples. (**C**) CRC classifier score on validation samples with ichorCNA values ≤ 3% tumor fraction, demonstrating that the 5hmC-based classifier maintains robust performance on samples with a low tumor fraction. The corresponding table presents the percentage of samples on either side of the classification threshold (0.5), demonstrating that the classifier performs similarly across CRC stages. (**D**) The top significant biological pathways identified that relate to the pathway relationship of genes regulated by enhancer features in the 5hmC classifier indicate a global immune response to tumorigenesis.

The 5hmC classifier was then applied to previously unseen samples from the validation set (220 CRC and 164 controls). Overall, the AUC in the validation set was 90% for CRC versus controls. The AUC was highest at stage 4 (94%) and declined only slightly to an AUC of 89% at stage 1 (Fig. 4A).

The 5hmC classifier achieved a specificity of 84%, with a sensitivity of 80% for CRC (78%, 83%, 75% and 93% for stages 1, 2, 3 and 4, respectively). The performance at stage 1 was comparable with other recently reported classifiers for colorectal cancer (Fig. 7C). In the validation samples, we detected CRC (all stages combined) with 55% sensitivity when specificity was fixed at 95%.

The 5hmC classifier reported in Fig. 4 was trained on a sample set that balanced age, ethnicity, sex and processing batch samples evenly across case and controls (Table 1). We trained another model that accounted for an extended set of clinical covariates that could affect epigenetic states (i.e., age, ethnicity, sex, diabetes, and use of statins, alcohol, tobacco and NSAIDs) via propensity score weighting of samples. This covariate-controlled classifier had an AUC of 90% in the training set and 85% in the validation set (Tables 3 and 4), suggesting that the classifier is not substantially confounded by covariates such as drug intake or comorbidities.

**Table 3:** Training performance of 5hmC classifiers for other cancers and conditions.

Condition	AUC (%)	Sensitivity at 90%	Sensitivity at 95%
CRC vs controls	90 (87–93)	71 (60–81)	55 (36–71)
CRC vs controls (weighted for additional clinical covariates)	90 (87–93)	72 (59–81)	59 (38–72)
CRC vs controls and adenomas	83 (80–86)	61 (46–73)	31 (23–44)
Lung cancer vs controls	80 (73–87)	37 (21–73)	18 (11–38)
Breast cancer vs controls	82 (75–89)	33 (22–56)	16 (11–29)
Prostate cancer vs controls	79 (71–88)	30 (20–54)	15 (10–28)
CRC vs other cancers	78 (74–82)	34 (26–47)	17 (13–24)

**Table 4:** Comparison of the validation performance of classifiers trained with different feature types.

Feature type	AUC (%)	Sensitivity (%)	Specificity (%)	Sensitivity at 95% specificity
CRC vs controls	90 (87–93)	81 (71–88)	84 (76–91)	55 (36–71)
CRC vs controls (weighted for additional clinical covariates)	85 (81–89)	75 (61–82)	82 (74–89)	45 (29–59)
CRC vs controls and adenomas	81 (79–87)	80 (73–86)	65 (52–77)	61 (42–71)

When adenoma samples (both individuals with nonadvanced and advanced disease) were added to the control samples for 5hmC classifier training, the AUCs in the training and validation sets were 83% and 81%, respectively, a poorer performance than the classifier trained on CRC and control samples only (Tables 3 and 4). When the original 5hmC classifier trained to detect CRC samples from controls was applied to the adenoma samples in the validation set (Table 5), 16% of nonadvanced adenomas and 22% of advanced adenomas were incorrectly classified as CRCs (compared to 17% of controls).

**Table 5:** The 5hmC classifier trained for CRC vs controls was applied to the validation data set. 95% CI computed using Binomial Proportions Test.

Disease	Stage	% CRC	95% CI	CRC	N
Advanced adenoma		21.95	(13.0, 31.0)	18	82
Arthritis		0.00	(0.0, 0.0)	0	7
Breast cancer	1	33.33	(0.0, 87.0)	1	3
Breast cancer	2	31.58	(11.0, 52.0)	6	19
Breast cancer	3	25.00	(0.0, 55.00)	2	8
Breast cancer	4	75.00	(33.0, 100.0)	3	4
COPD		50.00	(0.0, 100.0)	1	2
Colorectal cancer	1	77.78	(66.0, 90.0)	35	45
Colorectal cancer	2	85.06	(78.0, 93.0)	74	87
Colorectal cancer	3	75.41	(65.0, 86.0)	46	61
Colorectal cancer	4	92.59	(83.0, 100.0)	25	27
Control		17.45	(11.0, 24.0)	26	149
Lung cancer	1	42.86	(17.0, 69.0)	6	14
Lung cancer	2	68.75	(46.0, 91.0)	11	16
Lung cancer	3	71.43	(48.0, 95.0)	10	14
Lung cancer	4	85.71	(60.0, 100.0)	6	7
Non-advanced adenoma		15.91	(8.0, 24.0)	14	88
Ovarian cancer	1	83.33	(54.0, 100.0)	5	6
Ovarian cancer	3	100.00	(100.0, 100.0)	4	4
Prostate cancer	1	100.00	(100.0, 100.0)	1	1
Prostate cancer	2	54.55	(25.0, 84.0)	6	11
Prostate cancer	3	12.50	(0.0, 35.0)	1	8
Prostate cancer	4	80.00	(45.0, 100.0)	4	5
Stomach cancer	1	100.00	(100.0, 100.0)	1	1
Stomach cancer	2	100.00	(100.0, 100.0)	5	5
Stomach cancer	3	100.00	(100.0, 100.0)	2	2
Ulcers		16.67	(0.0, 46.0)	1	6
Bladder cancer	1	100.00	(100.0, 100.0)	5	5
Bladder cancer	2	100.00	(100.0, 100.0)	1	1
Bladder cancer	3	100.00	(100.0, 100.0)	1	1
Bladder cancer	4	100.00	(100.0, 100.0)	2	2

We trained 5hmC classifiers that successfully distinguished lung (AUC 80%), breast (AUC 82%), and prostate (AUC 79%) cancers from controls. We also trained a 5hmC classifier that distinguished CRC from these other cancers (AUC 78%) (Table 3), suggesting that it is possible to derive a 5hmC classifier that identifies CRC specifically rather than cancer more generally. When the original 5hmC classifier trained to detect CRC samples from controls was applied to the other cancer samples in the validation set (Table 5), the small numbers of stomach, bladder and ovary cancer samples were classified as CRC at a higher rate than actual CRC samples, while cancers of the breast and lung were classified as CRC less often than actual CRC samples.5hmC classifier is robust to low circulating tumor DNA as a proportion of cell free DNA.

### 5hmC classifier is robust under low ctDNA fraction

To further investigate the finding that the performance of the 5hmC classifier was similar across cancer stages, we estimated the amount of ctDNA in cfDNA samples in the validation set using the ichorCNA tumor fraction statistic. ichorCNA is reported to have a limit of detection of ~ 3% at a mean sequencing depth of 0.1x^36^.

At this threshold, we detected tumor fractions of 3–4% in the cfDNA of ~ 6% of control samples, with the remaining samples having a tumor fraction of < 3%. We interpreted this as a false positive rate of the ichorCNA method, although it is possible that a small number of “control” individuals could have undiagnosed non-CRC tumors, given the age range. Using the 3% threshold as a limit of detection, the 5hmC classifier correctly called 97% of CRC samples with a detectable tumor fraction and 75% of CRC samples with a tumor fraction below the 3% detection threshold (Fig. 4C). This demonstrates that the 5hmC classifier is robust when the tumor fraction is undetectable by the ichorCNA method.

Lowering the limit of detection to the median tumor fraction (1.4%) observed in the controls (implying that 50% of controls have detectable tumor fraction) still resulted in 76% of samples being classified correctly in the undetectable class (with 83% being classified correctly in the detectable class). In contrast to the higher (3%) limit of detection at this level, CRC classification and tumor fraction class (detectable/undetectable) were statistically independent (Fisher’s exact test for dependence was non-significant). As expected, the mean ctDNA fraction was greater in the CRC samples than in the controls (4.6% versus 1.8%, Mann–Whitney U test p = 0.003), and ctDNA fractions were correlated with the reported cancer stage (Spearman’s rho = 0.25, p = 0.0002, Fig. 4B). Not only did the 5hmC classifier correctly call samples with a higher tumor fraction (Supplementary Fig. 1), but it was also highly robust for samples where the tumor fraction was below the ichorCNA detection threshold of 3% (Fig. 4C). Therefore, the 5hmC classifier is not solely dependent on the ctDNA fraction.

### Interleukin signaling may drive 5hmC-based classification

To investigate the biological signals driving the 5hmC classifier’s performance in CRC samples with a low ctDNA fraction, the classifier’s feature enhancers were mapped to gene names and queried in Key Pathway Advisor software (Clarivate). IL11 signaling to the PIK3CA cascade was the top ranked pathway. This indicates that cfDNA fragments from the immune system may drive detection (Fig. 4D, Supplementary Fig. 2). We further assessed features identified from training a classifier using only early-stage CRC samples (stages 1 and 2) and compared these with the features from a classifier trained only on late-stage CRC samples (stages 3 and 4). We found that the interleukin signal is present in late-stage CRC. Evidence pointed to an association with microRNAs in early-stage CRC (Supplementary Fig. 3).

### Performance of a region-based fragmentomics classifier has greater dependence on cancer stage and ctDNA fraction than a 5hmC classifier

To investigate the apparent lack of stage dependence observed for the 5hmC classifier, we interrogated the cfDNA fragment characteristics from the same samples (the validation set). These fragmentomic characteristics were observed from the input libraries generated alongside hydroxymethylome capture. Therefore, the data were readily available from exactly the same sample set but without information about 5hmC levels. Fragmentomics has previously been reported to be stage dependent. Using a technique similar to the DELFI method (“Supplementary Methods”), in silico analysis of read depth and estimated DNA fragment size was performed on sequencing reads from the input libraries, comparing the number of long to short fragments in 5 Mb windows.

A classifier was produced using the same machine learning methodology as that used for the 5hmC classifiers. This fragmentomics classifier distinguished CRC samples from controls with an AUC of 83% and 62% sensitivity at 95% specificity in the validation set.

**Figure 5:**
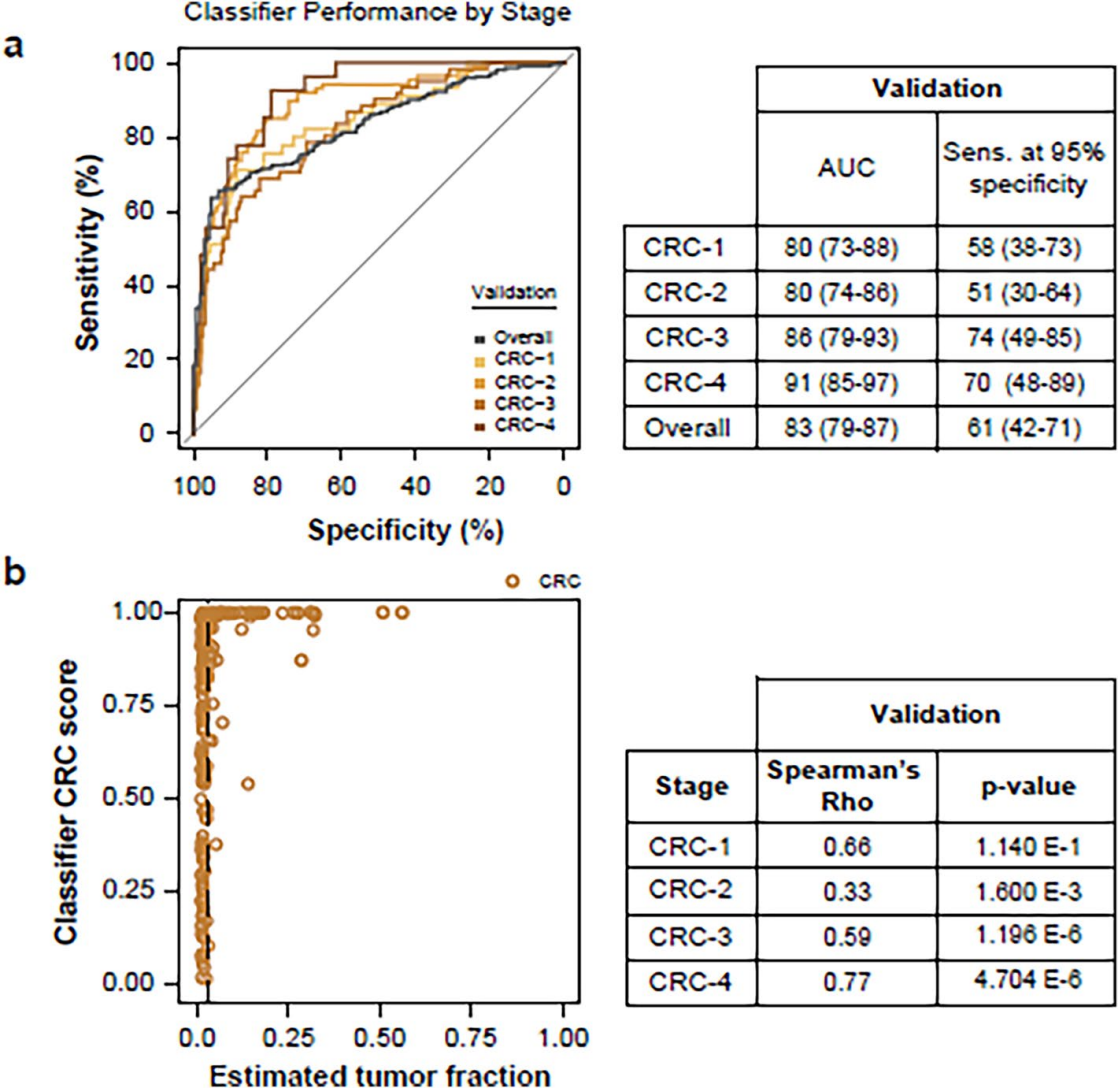
(**A**) Classifier trained using a DELFI-like approach demonstrates CRC stage-dependent performance versus controls in validation samples. Cut-of-values are reported in Supp. Table 1. (**B**) The classifier prediction probability shows strong concordance with the estimated tumor fraction (ichorCNA), particularly in late stages in both training and validation samples.

The performance of the fragmentomics classifier decreased from 91% for stage 4 CRC samples to 80% for stage 1 samples (Fig. 5A). This represents a higher loss of performance to detect early-stage CRC than the 5hmC classifier, which retained performance at an early stage (Fig. 7A,B). The correlation between AUC and CRC stage was higher for the fragmentomics classifier (Spearman’s rho = 0.95, *p* = 0.05) than for the 5hmC classifier (Spearman’s rho = 0.80, p = 0.333). In addition, the fragmentomics classifier score was more highly correlated with ctDNA content in late-stage tumors (stage 4 Spearman’s rho = 0.77, p = 4.7 × 10^–6^) than the 5hmC classifier (Spearman’s rho = 0.66, p = 0.00028), potentially explaining the fragmentomics performance gain in these late tumors (Fig. 5B).

**Figure 6:**
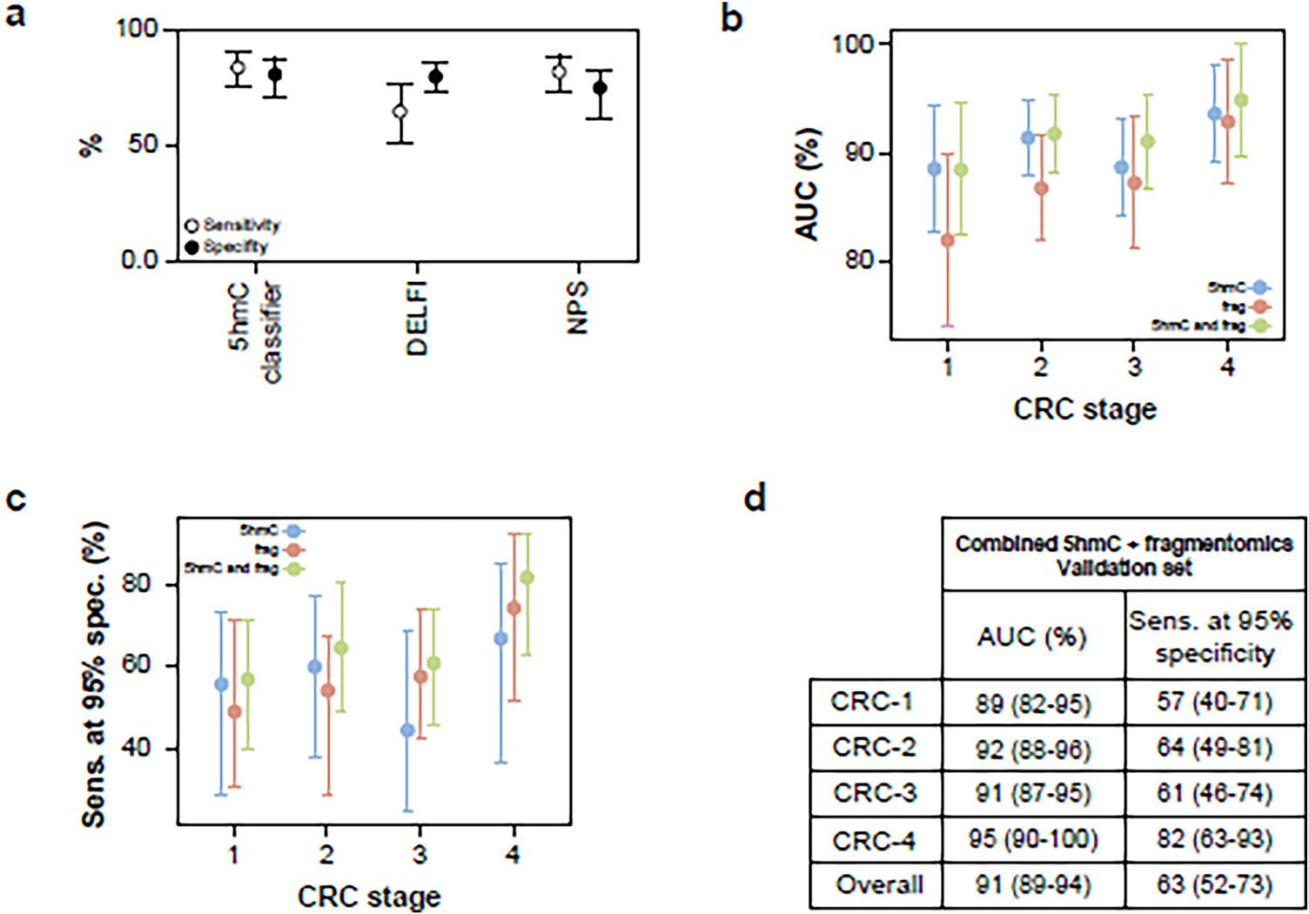
(**A**) Overall, the median performance estimate was higher for the 5hmC classifier than for the DELFI and NPS classifiers. (**B**–**D**) Median AUC and sensitivity at 95% specificity of 5hmC, DELFI-like fragmentomics approach and combined classifier. The 5hmC classifier performs better than the DELFI-like fragmentomics classifier in early CRC stages (1 & 2), while in late stages (3 & 4) 5hmC shows significant additivity at higher specificity (95% specificity).

We also trained a classifier with features based on the positioning of nucleosomes using the Nucleosome Presence Score (NPS) method; see “Supplementary Methods”. We demonstrated that the enhancer-based 5hmC classifier (median AUC 90.3%) outperforms both the fragmentomics (median AUC 83.1%) and NPS classifiers (median AUC 85.2%) across all CRC stages in terms of sensitivity and specificity (Fig. 6A–C, Table 4).

### 5hmC is additive to orthogonal sample characteristics

In an effort to capture all information yielded during sample processing, we trained a classifier including genome-wide 5hmC data, genome-wide region-based fragmentomics data (as above), and further sample characteristics such as library yield, genome-wide fragment size distribution and copy number-related quantities. The performance of the resultant classifier to detect CRC in the validation set increased to an AUC of 91% and 63% sensitivity at 95% specificity (Fig. 6B–D This gain in performance was evident in stages 2–4 of CRC compared to a classifier trained using 5hmC data alone).

## Discussion

Patients diagnosed with early-stage CRC have markedly better survival than patients diagnosed with late-stage CRC^37^. Detection of early-stage CRC is challenging due to low tumor DNA content in blood. Here, we report that profiling of 5hmC in cfDNA is powerful for early CRC in liquid biopsy.

**Figure 7:**
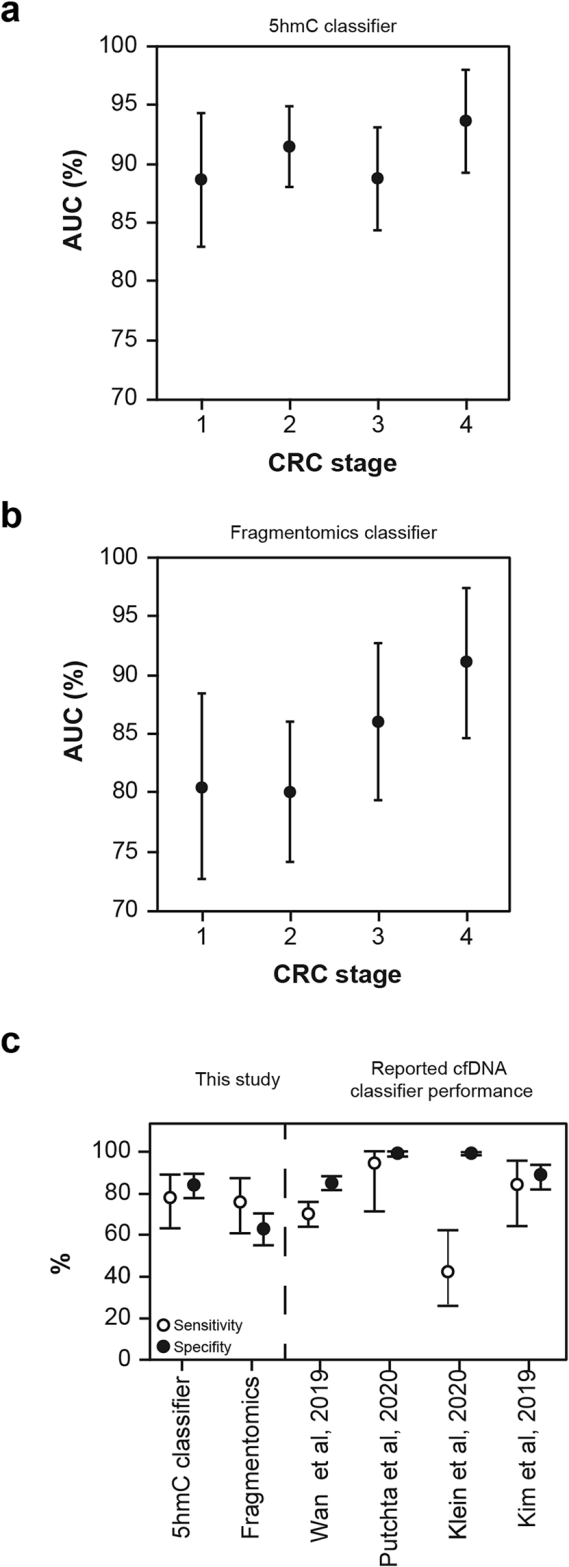
(**A**, **B**) A classifier trained on 5hmC levels in enhancer regions maintains performance at early-stage cancer(CRC Stage vs AUC: Spearman’s rho = 0.80, p = 0.333) compared to a model trained on cfDNA fragment size and coverage (DELFI-like approach) (CRC Stage vs AUC: Spearman’s rho = 0.95, *p* = 0.05). (**C**) The 5hmC-based classifier performs comparably to reported classifiers for stage 1 CRC. To gain approximately comparable confidence intervals, 95% binomial confidence intervals were computed for all classifiers using publicly available information^8,12,33,34,38^. The CRC classifier from Putcha et al. contains both Stage 1 and Stage 2 samples.

The 5hmC classifier reported here detected stage 1 CRC with an AUC of 89% and a sensitivity of 56% at 95% specificity. The operational performance (classifying each sample in the validation set without fixing specificity) on the validation set for stage 1 CRC (78% sensitivity at ~ 85% specificity) is statistically equivalent to several other reports using both non-5hmC and 5hmC-based classifiers for mixed stages of CRC, including later stage^8,12–15,29^ (Fig. 7C).

Four examples are included in Fig. 7C, including training and validation performance from a study using read counts in cfDNA whole genome sequencing^12,34^ and two studies reporting validation performance of 5mC levels^33,38^. Wan et al. reported that their classifier based on cfDNA read counts performed at a mean AUC of 92% (95% CI 0.91–0.93)^12^ in cross validation within a training set (n = 817 samples). In further work from the same group, Putcha et al. reported validation (not yet peer reviewed) of the classifier in a small validation set (n = 17) with a mix of stage 1 and 2 samples^34^. We include both the training and validation reports in Fig. 7C, as it demonstrates how a classifier that performs very similarly to the one described here (with a similar training regime designed to emphasize bias reduction) may be tuned for higher specificity with a resulting trade-off with sensitivity. Liu and colleagues report a classifier based on methylation in cfDNA. In the validation set (n = 610), this classifier achieved a specificity of 99.3% (95% CI 98.3–99.8%), but the sensitivity was just 54.9% across a range of cancer types and stages^8^. The performance of this classifier in detecting cancer (types and stages) was recently reported as 51.5% assessed in a large validation set (n = 2823 cancer patients of different types and stages [of whom 206 had CRC] and n = 1254 controls)^38^. In stage 1 CRC, the sensitivity was 43.3% at 99.5% specificity^38^. The cfDNA 5mC-based classifier reported by Kim et al. correctly classified 94% of CRC samples (stages 1–3) with 94% specificity in a validation set (n = 72 CRC and 35 controls)^33^.

Li et al. used a cfDNA 5hmC classifier to detect CRC with an AUC of 94% (88% sensitivity, 89% specificity) in a small validation population (69 subjects)^14^. Guler et al. used 5hmC to detect pancreatic cancer, achieving an AUC of 92–94% in two independent validation sets comprising 228 and 17 subjects^15^.

Our genome-wide approach to cfDNA profiling allowed selection of the most relevant 5hmC-enriched genomic regions on which to build a classifier, perhaps including signals not carried within ctDNA. The performance of our 5hmC classifier did not appear to be dependent on a high ctDNA fraction in blood, even in cancer samples where the established ichorCNA method was unable to detect ctDNA. Notably, this pattern of sensitivity for early-stage cancer held across all the cancer types on which we tested the classifier (Table 5). We hypothesize that the genome-wide 5hmC signal included a signal from cfDNA arising from non-cancerous cells that undergo state changes in response to tumorigenesis and progression. This is supported by the finding that the classifier using 5hmC enhancer regions was significantly enriched in inflammatory immune responses such as interleukin signaling. Indeed, ~ 55% of cfDNA derives from white blood cells, according to whole genome analysis^39^.

## Limitations

Although we and others have reported results at high specificities, we interpret performance at these levels cautiously due to the comparatively small population of cancer-negative individuals that is unlikely to fully account for sample heterogeneity, clinical and demographic biases present in an asymptomatic CRC screening population.

Another limitation was the possibility of selection bias in the study population since blood samples were collected from individuals presenting for colonoscopy. None of the clinical covariates we tested impacted classifier performance (Table 1), but not all possible risk factors were collected, so we cannot exclude the effects of some covariates on classifier performance.

## Future work

We show that the CRC classifier is sensitive to stomach, bladder, prostate and ovarian cancers, although the classifier has higher CRC specificity with respect to breast cancer stages 1–3 (Table 5). This demonstrates that the 5hmC classifier retains a general cancer signal. However, we note that it is also possible to train a classifier to distinguish CRC from other cancers (Table 3), suggesting that there is a cancer-specific 5hmC signal, and further work could include optimization of the classifier training program to improve CRC specificity.

Overall, these results demonstrate that changes in 5hmC DNA modifications hold promise for detecting early-stage cancer.

## Conclusions

The 5hmC classifier successfully detected CRC samples in an independent validation set, regardless of cancer stage, and was robust to covariates and comorbidities that may also influence the epigenome.

In conclusion, this is the largest study to date to demonstrate the power of 5hmC to detect early-stage CRC via blood cfDNA in a heterogeneous, well-balanced, well-powered cohort, employing an internal validation set to verify performance. We conclude that 5hmC is a powerful biomarker that can be used to enhance noninvasive diagnostic tools for the detection of early-stage and treatable cancer.

## Methods

### Study design

This multicenter, case–control study aimed to create and assess a 5hmC-based classifier for detecting CRC, even at an early stage. Blood samples were obtained from 2483 individuals from 12 commercial suppliers covering 56 individual sites, sourced from both biobanks (~ 40%) and prospective collection (~ 60%). For each of the 12 vendors approvals were obtained from the relevant committee, namely: Capital Biosciences, Inc, 900 Clopper Rd, Suite 120 Gaithersburg, MD, 0878, USA; Quorum Review Investigational Review Board, DxBioSamples, Inc 11199 Sorrento Valley Rd., Suite 206, San Diego, CA 92121, USA; Diagnostics IRB, iProcess Global Research Inc, 1135 Kinwest Pkwy, Suite 150, Irving, TX 75063, USA; Society for Medical & Health Care Research Independent Ethics Committee and Italian Institutional IRB-waiver (details available on request); National BioService LLC, 17A Litovskaya str., 194100, Saint Petersburg, Russia; Independent Ethics Committee of Arte Med Assistance, LLC, Proteogenix, 15 rue de la Haye, 67300 SCHILTIGHEIM France; N. N. Blokhin NMRCO MHRF, Discovery Life Sciences, 800 Hudson Way, Suite 1700, Huntsville, AL 35806, USA; WCG-IRB provided research oversight, Trans-Hit Bio, 3090 le Carrefour Boulevard, Suite 750, Laval, Quebec H7T 2J7, Canada; MIHS IRB, Greater Manchester Central REC, Oschner Clinic Foundation IRB and Comite de Etica en Investigation del Hospital la Mision SA de CV, Geneticist Inc, 520 West Colorado Street, Glendale, California, 91204, USA; Kjarkiv National Medical University Ethics Committee, iSpecimen Inc, 450 Bedford Street, Lexington, MA 02420, USA; Quorum Review IRB, Precision for Medicine, LLC, 10 Commerce Way # C2, Norton, MA 02766, USA; Hanoi Medical University IRB, Reprocell USA Inc, 9000 Virginia Manor Road, Suite 207, Beltsville, 20705, USA; Western IRB and Tissue Solutions Ltd, Ground Floor, Fleming Pavilion, Todd Campus, West of Scotland Science Park, Glasgow, G20 0XA; Diagnostics Investigational Review Board.

Control samples were from people aged 45–85 years who were at average risk for CRC and had been assessed by colonoscopy with results that showed no presence of CRC or adenomatous polyps (adenomas). Cancer samples were obtained from people aged 45–85 years who underwent colonoscopy and were diagnosed with CRC, lung, breast, bladder, prostate, ovarian, stomach cancer or adenomas. Advanced adenoma was defined as high-grade dysplasia or with ≥ 25% villous histologic features OR measuring ≥ 1 cm in the greatest dimension, in agreement with Imperiale et al.^40^. Inclusion/exclusion criteria are available in Supplementary Materials 2.

We recorded the following data for each blood sample donor: age, sex, ethnicity, smoking, diabetes status, previous medical history, concomitant medications (including daily NSAID use) and type of diagnosis.

Extraction of cfDNA was attempted from double spun plasma donated by 2483 individuals (Fig. 1). cfDNA from 2198 individuals was passed forward to sequencing. 5hmC was quantified across the cfDNA genome for all individuals via comparison of region read depth in sequencing libraries enriched by highly sensitive capture of 5hmC to a nonenriched library. The hydroxymethylome of 701 participants was interrogated by machine learning algorithms to produce classifiers distinguishing CRC from a range of other conditions. The classifiers were then tested on previously unseen 5-hydroxymethylome data from 691 other individuals (the validation set).

The sample size of the validation set was computed based on demonstrating improvement over the multitarget stool DNA test (sensitivity 92.3% and specificity 86.6% [95% CI 85.9–87.2%]) and fecal immunochemical test (FIT) (sensitivity 73.8% and specificity 94.9% [95% CI 94.4–95.3%])^40^. Achieving a desired 1-sided sensitivity for CRC, we initially chose a lower bound of 83.0% for the confidence interval and a point estimate of 92.3% for this study. Without accounting for gender, age and stages of disease differences, 95% power, 0.025 alpha and 5% dropout rate, a sample size of at least 330 CRC confirmed cases was deemed required for sensitivity characterization. To achieve approximately the same power, a similar sample size of at least 340 would be required to demonstrate the desired specificity. However, we chose to trade off model training error with validation error, reducing the validation sample size to 220 CRC and 164 controls. Consequently, the ability of 5hmC to detect CRC may be underestimated by this study.

The study was performed in accordance with the Declaration of Helsinki and was approved by the relevant independent ethics committee or institutional review board for each commercial supplier of blood samples. Written informed consent was obtained from all donors of samples.

### Method details

#### Blood sample collection

Where possible, sampling was performed before colonoscopy. Blood (10 ml) was drawn into a K2 EDTA blood tube, placed on ice and processed within 4 h. Samples were centrifuged (2000*g* for 10 min at room temperature). The plasma layer was transferred to a clean tube and was again centrifuged (2000*g* for 10 min at room temperature) to remove any remaining cellular material. Double-spun plasma was aliquoted into tubes in at least 1 or 2 ml volumes and then immediately frozen and stored at − 80 °C before shipment to the central laboratory for investigation.

#### Sample balancing

To avoid confounding the biological signal, the OSAT algorithm^41^ was utilized to achieve an even distribution of disease state and potential confounders across experimental plates for both cfDNA extraction and hydroxymethylome capture. The associations of these factors with batch were tested using a chi-square test, and the design was modified where necessary. The distribution of disease, sex, ethnicity, and age group did not show statistically significant variation (p < 0.05) over the 96-well plates that were subject to automated library processing. This ensured that any plate-related processing biases were distributed evenly across sample characteristics.

#### cfDNA extraction and library creation

cfDNA was extracted using the NextPrep-Mag™ kit on the Chemagic Prime platform (Perkin Elmer Chemagen Technologies GmbH, Baesweiler, Germany) using 2 ml of plasma in 48-well plates. Two plates were extracted simultaneously and combined in a single 96-well plate at the end of the extraction process. cfDNA concentration was pre-quantified by PicoGreen (Life Technologies) assay on a CLARIOstar plate reader. cfDNA that reached a threshold concentration by PicoGreen was further quantified and assessed for cfDNA purity by gel electrophoresis (Fragment Analyzer, Agilent, Santa Clara, CA, USA). cfDNA samples with a yield ≥ 5 ng were normalized, and 5 ng was plated using the Chemagic Prime instrument into 96-well plates ready for library preparation. Five 166 bp synthetic controls were included in every sample of the experiment to control the quality of hydroxymethylome capture. The positive controls contained 1, 3 or 6 5hmC residues, and the negative controls contained 6 5mC residues and unmodified Cs residues.

cfDNA samples were end repaired, adenylated (Kapa Hyper Prep kit, Roche Sequencing and Life Science), ligated to unique dual index (UDI) adaptors (Illumina TruSeq DNA Unique Dual (UD) Indexes, Illumina, San Diego, CA, USA) and purified using SpeedBeads™ magnetic carboxylate-modified particles.

Part of each sample (1 μl) was used to create an “input library” by directly PCR amplifying (9 cycles) ligated cfDNA. The remaining 12 μl of each sample was used for hydroxymethylome capture.

#### Hydroxymethylome capture

After adapter ligation, the cfDNA strands were denatured and copied using a primer complementary to the sequence in the Illumina adapter using a DNA polymerase I Klenow fragment (3′ → 5′exo) (Enzymatics, QIAGEN). Consequently, all the DNA fragments in the library comprised duplexes where one strand represented the original native genomic DNA, complete with epigenetic marks, and the other strand was an unmarked complimentary copy. 5hmC residues in the original genomic strand were selectively labeled with azide-modified UDP-Glucose by incubation with UDP-6-N3-Glu (Jena Bioscience, Jena, Germany) and T4-beta-glucosyltransferase (Thermo Fisher Scientific, MA, USA). In turn, the azide groups were biotinylated with DBCO-PEG4-Biotin (Click Chemistry Tools, AZ, USA).

Samples were then purified using the DNA Clean & Concentrator kit (Zymo Research, Irvine, CA, USA). The 5hmC biotin conjugates were selectively bound to streptavidin beads (Dynabeads M-270, Invitrogen, Carlsbad, CA, USA). Finally, the single strand copies of the hydroxymethylome library were liberated from the beads by 0.1 M NaOH and PCR amplified (16 cycles). (Fig. 2A). The input and hydroxymethylome libraries were purified using SpeedBeads™ magnetic carboxylate-modified particles (Sigma-Aldrich).

Concentration was determined using PicoGreen, and library size and concentration were also determined using Fragment Analyzer data.

#### Sequencing

We prepared 3 nM of non-hydroxymethylome enriched libraries (“input”) and hydroxymethylome library pools. Libraries were sequenced on the NovaSeq platform using 100 bp paired-end mode, yielding approximately 60 million reads per sample.

#### Bioinformatic data processing and quality control

Demultiplexing and trimming were achieved using bcl2fastq Read (Illumina Basespace). Reads were aligned to the human genome (GRCh38) using BWA-MEM^42^, and those with a BWA mapping quality score (MAPQ score) less than 1 were filtered. Sequence duplicates were removed using Picard MarkDuplicates. Libraries were scored for quality on multiple criteria. A cumulative quality score of 0 indicates perfect library quality and the absence of quality issues. Libraries scoring over 15 were discarded (along with their mate pair). Libraries scored 5 points if they had < 10 M reads post deduplication, or a ratio of reads per kilobase of gene body, per million mapped reads (RPKM) across all for gene bodies divided by the RPKM in intergenic regions of < 1 (for the hydroxymethylome libraries only), or a lack of spike-in control amplification (< 1 for ratio of hydroxymethylation over methylation and cytosines), or a mitochondrial RPKM > 1000, or < 10% of reads mapping to peaks, or a median insert size > 200 nt, or uniformity < 0.8 (for input libraries only). Libraries scored 3 points if they had > 1.5× the interquartile range for 26 quality metrics. Hydroxymethylome libraries scored 1 point if they deviated by > 2 standard deviations from ranges of gene body versus intergenic enrichment, duplication rate and coverage of the previously observed in other in-house studies and to input libraries that deviated by > 2 standard deviations in sequence diversity score to the observed ranges from previous studies.

#### Feature definition

To calculate 5hmC levels at gene enhancers, we first calculated read counts using Bam readcounts v0.01. RPKM was calculated over candidate gene enhancers (adapted from) downloaded from GeneCards v4.4. 5hmC enrichment was computed as the log2 ratio between the hydroxymethylome library RPKM and the input library RPKM after the inclusion of a pseudocount of 1.

We produced a set of cfDNA fragment features inspired by the DELFI approach adapting the computational methodology, available via GitHub^43^. Briefly, we divided the genome into 100 kb bins and quantified cfDNA fragment sizes per bin. We removed blacklisted regions, genomic gaps (UCSC table) and nonstandard chromosomes a priori. We excluded outlier bins in fragment size, retaining only fragments between 100 and 220 nt in length. Finally, we split the genome into 100 kb bins (in total 26,170 non-overlapping genomic regions) and calculated the following characteristics of fragment size distribution per genomic bin: number of short fragments (100–150 nt), number of long fragments (151–220 nt), ratio between short and long fragments and the total number of fragments. This approach generates 26,170 features per metric and per sample. The last step is the averaging of the 100 KB bins into larger non-overlapping genomic regions of 5 MB (in total 512 bins).

A second set of fragmentomics features, referred to as Nucleosome Presence Score (NPS) features, consists of metrics related to nucleosome presence and captures information at a highly localized scale. Our approach is inspired by the windowed protection score method of Snyder et al.^16^ but includes some key differences. First, 40 samples (20 CRC and 20 controls) from the training cohort were reserved for developing the NPS approach. Subsequent models were never trained using these 40 samples. Based on 5hmC pulldown libraries in these 40 samples, a total of approximately 235,000 regions were identified by merging peaks produced by the MACS2 and EPIC2 peak callers. Using the bedtools coverage tool, average per-base coverages were computed for each region for each of the 40 samples. By sorting regions by the median coverage across all 40 samples, the 200 regions with the highest median coverage were chosen. In total, these regions covered approximately 4.5 Mb of the genome. Next, the reads from the input libraries of the 40 samples were pooled, thus producing a single .bam file of depth 110.65×. This pooled sample was used to identify nucleosome positions in the 200 regions defined above. Nucleosome calling was performed by computing NPS profiles and using a simplistic peak calling approach. This approach assigned nucleosomes to NPS maxima in a 151 bp sliding window. If multiple maxima existed within 76 bp of each other, they were assumed to represent a single nucleosome position located at the midpoint between the maxima. NPS profiles were computed using fragment size data describing the start and end positions of fragments. Fragment data were generated from deduplicated bam files with non-properly paired reads removed using Samtools (− f2 flag). Pairs of reads were collapsed into fragments using the bedtools bamtobed command, and fragments of length more than 1000 bp were removed, which were assumed to be errors. To compute the NPS profile in a given region, a sliding window approach was employed with a window size of 121 bp and with NPS values defined for the midpoint of the window. To capture single nucleosome configurations, fragments less than 120 bp or larger than 250 bp were discarded. For each window, the NPS was defined as the ratio of the number of fragments spanning the window (n_span) to the number of fragments with at least one endpoint inside the window (n_within). As a result, the metric takes positive values and is independent of read depth. To limit cases of divergence, a pooling approach was applied wherein the NPS at position i was defined using information from ± 5 neighboring positions:

$$\sum\limits_{{i - 5}}^{{i + 5}} {n_{{within}} } = 0$$

In events where the NPS was set as NA and subsequently imputed. Imputation was achieved using a simple “fill in the gaps” strategy where missing values were assigned to linearly bridge the nearest non-NA values. Samples where more than 90% of the NPS profile of a given region was NA were categorized as “undefined” for this specific region. Such incidences were addressed by the feature-level imputation strategy. No feature had more than 2% missingness. Finally, to construct a clear nucleosome signal, NPS profiles were smoothed using a Savitzky-Golay filter of degree 2 with a window size of 151 bp.

Feature matrices were constructed for all samples using the nucleosomes identified from the 40 left out samples. Features were defined as the minimum NPS value in a ± 50 bp neighborhood around the midpoint between two nucleosome positions, provided the nucleosomes were no more than 300 bp apart. The midpoint between nucleosomes was found to be marginally more informative than the actual nucleosome positions.

#### Classifier development and internal cross-validation

We trained support vector machine (SVM) models using a linear kernel function on feature-scaled (z-score normalization) 5hmC levels of enhancers quantile-normalized over samples (see Supplementary Fig. 4). An ensemble of 50 models were trained. Each model in the ensemble was trained on a randomly selected 80% of the samples in the training set. The trained models were used to predict 20% of the remaining samples in an internal cross-validation procedure within the training set. We identified features significantly correlated with technical covariates such as age, sex and vendor using linear regression and ANOVA F-test for continuous and categorical variables, respectively. These features were then excluded. Model hyperparameters (the C parameter) were optimized for the highest AUC within a tenfold cross validation strategy. Performance was averaged across the 50 individual learners, and the unique features selected by all 50 were retained (Supplementary Fig. 4). The 95% confidence intervals of various performance metrics (e.g., AUC, sensitivity) for each of the classifier ensembles were computed using 2000 bootstrap replications of predicted samples. Classifier development and cross-validation within the training set were performed by the model development team, who logged trained and timestamped classifiers in a registry, along with auxiliary information on the training procedures for each model. An MD5 checksum was then computed for each classifier, functioning as a unique identifier.

#### Assessing classifier performance in the validation set

The cross-validated classifier was then assessed in the “held-out” validation set of 691 samples locked prior to the model development process.

To facilitate a blinded model validation strategy, a separate team performed the model validation. Version history on the classifier registry verified that hash keys had not been modified since initial logging. The validation team generated feature matrices and metadata files for the validation set and subsequently applied the models to the validation data. The validation team operated on virtual machines and storage belonging to a separate cloud project that was inaccessible to the model development team. During the model validation process, the hash key from each applied model was compared to the logged hash key to ensure model integrity. Here again, prediction probabilities from every learner within the classifier ensemble were averaged to compute the final prediction probability for each sample. Final performance metrics, such as the AUC, sensitivity and specificity, were computed based on the averaged prediction probabilities. The performance results were automatically uploaded to a cloud database without any intervention from the model development team.

#### Assessing tumor fraction with ichorCNA

We ran the ichorCNA workflow on input libraries sequenced for the internal validation sample set.

This involved first running readCounter from the hmmcopy version 0.1.1 with the following command with the parameters window size set to 1,000,000 and quality set to 20. We then further ran ichorCNA using the recommended settings for low tumor fraction samples as per below:

- centromere GRCh38.GCA_000001405.2_centromere_acen.txt \
- estimateNormal True -estimatePloidy True -estimateScPrevalence False \
- scStates 'c()' -txnE 0.9999 -txnStrength 10,000 -normal 'c(0.95, 0.99, 0.995, 0.999)' \
- ploidy 'c(2)' -maxCN 3 -normalPanel HD_ULP_PoN_hg38_1Mb_median_normAutosome_median.rds
- chrs 'c(1:22)' -chrTrain 'c(1:22)'
- gcWig $gc_hg38_1000 kb.wig

#### Functional analysis of 5hmC enhancer regions driving classifier performance

To develop a better mechanistic understanding of the classifier, we ran pathway analysis using the Key Pathway Analysis (KPA) tool on top discriminatory enhancers. Enhancers were ordered based on their average contribution to the classifier (averaged across the models), and from the top 500 enhancers that appeared in at least 5 individual models were selected for the pathway analysis. The top scoring gene target for each 500 enhancers was taken from the ‘connected_gene’ field in the GeneHancer database^44^ and used as the input for pathway analysis.

## Supplementary Information

Supplementary Information 1.

Supplementary Information 2.
