# Supplementary material for: Hydroxymethylation profile of cell-free DNA is a biomarker for early colorectal cancer
Source: Sci Rep. 2022 Oct 4;12:16566. doi: 10.1038/s41598-022-20975-1 (PMC9532421; doi:10.1038/s41598-022-20975-1)
Supplement: Supplementary file 1 — Supplementary Information 1. [file 41598_2022_20975_MOESM1_ESM.pdf]

# Hydroxymethylation profile of cell free DNA is a biomarker for early colorectal cancer

Supplementary Figures 1-4
Supplementary Table 1

Supplementary Figure 1.

Tumour Fraction and CRC prediction **(A)** Validation samples with increased tumour fraction have higher CRC scores **(B)** CRC classifier score on validation samples with ichorCNA values > 3% tumor fraction, demonstrating that the 5hmC based classifier maintains robust performance on samples across tumour fraction. The corresponding table (lower panel) presents the percentage of samples either side of the classification threshold (0.5) demonstrating that the classifier performs similarly across CRC stage

(A)

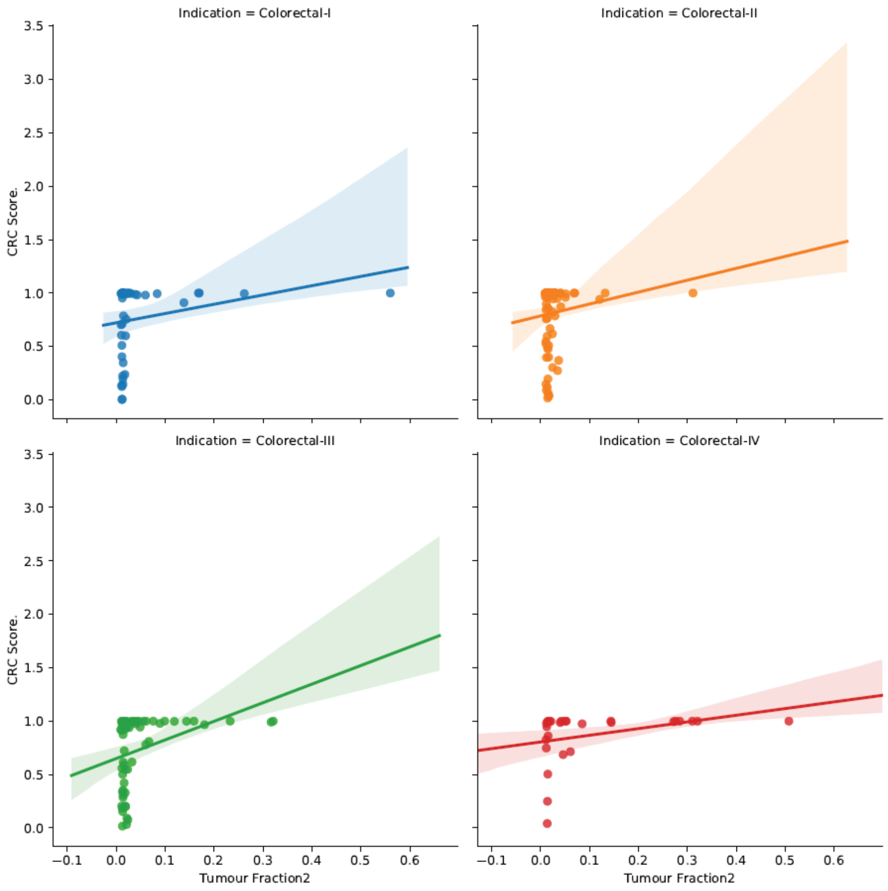


(B)

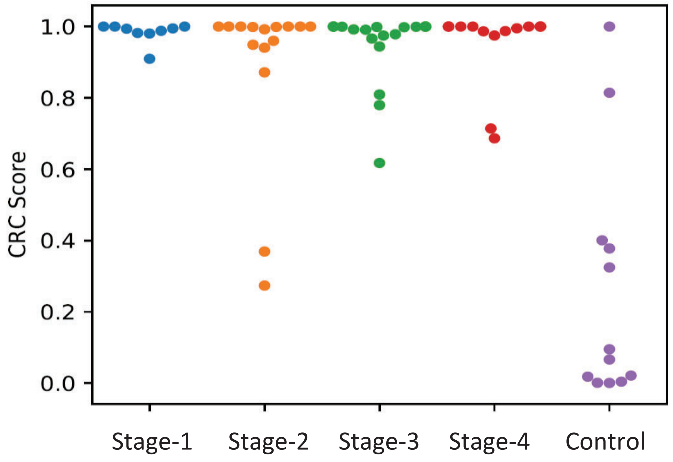


| | < 0.5 | ≥ 0.5 |
|---|---|---|
| Colorectal-I | 0.00 | 100.00 |
| Colorectal-II | 13.33 | 86.67 |
| Colorectal-III | 0.00 | 100.00 |
| Colorectal-IV | 0.00 | 100.00 |
| Control | 83.33 | 16.67 |

## Supplementary Figure 2

Immune response IL11 signalling pathway via MEK/ERK and PI3K/AKT cascades collated by Clarivate KPA, significantly enriched for genes whose enhancer 5hmC levels are implicated in CRC detection

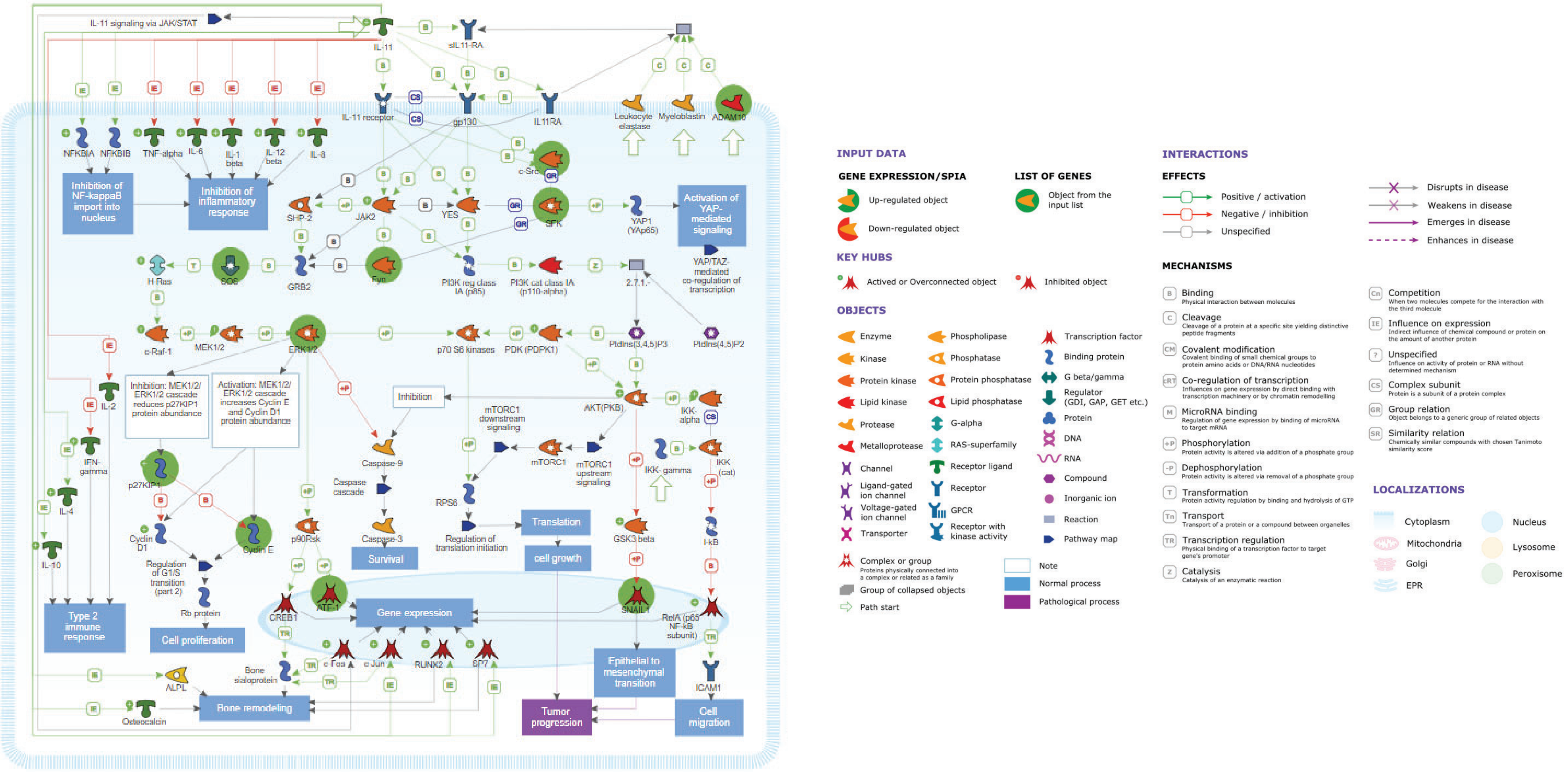

## Supplementary Figure 3

Early stage CRC genomic features show microRNA from tumour and its microenvironment. With both tumour and inflammatory signals adding to signal in later stages. KPA analysis of pathway maps and key genehubs from enhancer 5hmC levels that discriminate CRC stage 1 and stage 2 from control samples ("Early Stage"), and CRC stage 3 and 4 from control samples ("Late Stage"). Early stage pathway maps show a lack of interleukin related pathways, and return a microRNA role in CRC. Late Stage pathway maps include more immune related signal. Both early and late stage possess microRNA key gene hubs, implicating microRNA as a source of signal both in early and late stages of cancer.

### Top Pathway Maps

**Early Stage**

| # | Name | Input Data p-value | Key Hubs p-value | Union p-value ▲ |
|---|---|---|---|---|
| 1 | Epithelial cell anoikis in COPD | 9.935E-5 | 0.008453 | 2.332E-6 |
| 2 | Development_HGF signaling pathway | 0.004634 | 0.00689 | 1.296E-4 |
| 3 | TGF-beta 1-induced transactivation of membrane receptors signaling in HCC | 0.00629 | 0.008129 | 2.1E-4 |
| 4 | Cytoskeleton remodeling_Reverse signaling by Ephrin-B | 0.01222 | 0.00353 | 2.244E-4 |
| 5 | Development_FGFR signaling pathway | 0.007249 | 0.008783 | 2.626E-4 |
| 6 | Cytoskeleton remodeling_FAK signaling | 0.00886 | 0.009807 | 3.605E-4 |
| 7 | Main pathways of Schwann cells transformation in neurofibromatosis type 1 | 0.005784 | 0.01881 | 3.774E-4 |
| 8 | Role of microRNAs in cell migration, survival and angiogenesis in colorectal cancer | 0.01848 | 0.01482 | 0.001153 |
| 9 | Main chemotherapy drugs and their action in SCLC cells | 0.04422 | 0.02476 | 0.004619 |
| 10 | Development_The role of GDNF ligand family/ RET receptor in cell survival, growth and proliferation | 0.04578 | 0.02528 | 0.004882 |

**Late Stage**

| # | Name | Input Data p-value | Key Hubs p-value | Union p-value ▲ |
|---|---|---|---|---|
| 1 | PDE4 regulation of cyto/chemokine expression in inflammatory skin diseases | 8.841E-5 | 9.407E-6 | 2.465E-8 |
| 2 | Role of IL-8 in melanoma | 1.634E-5 | 5.041E-4 | 2.133E-7 |
| 3 | Glucocorticoids-mediated inhibition of pro-constrictory and pro-inflammatory signaling in airway smooth muscle cells | 7.493E-4 | 1.198E-4 | 2.305E-7 |
| 4 | Lysophospholipid mediators-induced inflammatory signaling in normal and asthmatic airway epithelium | 0.001193 | 1.948E-4 | 6.291E-7 |
| 5 | Signal transduction_Adenosine A3 receptor signaling pathway | 5.506E-4 | 0.01065 | 1.332E-5 |
| 6 | Influence of multiple myeloma cells on bone marrow stromal cells | 0.001582 | 3.587E-4 | 1.62E-5 |
| 7 | Apoptosis and survival_HTR1A signaling | 7.493E-4 | 0.01275 | 2.212E-5 |
| 8 | GLP-1 in beta cell apoptosis in type 2 diabetes | 7.493E-4 | 0.01275 | 2.212E-5 |
| 9 | Proteases and EGFR-activated mucin production in airway epithelium in COPD | 0.005837 | 0.001399 | 2.212E-5 |
| 10 | G-protein signaling_Proinsulin C-peptide signaling | 8.262E-4 | 0.0135 | 2.596E-5 |
| 11 | Development_GM-CSF signaling | 9.087E-4 | 0.01427 | 3.035E-5 |
| 12 | Signal transduction_PKA signaling | 0.03976 | 1.465E-4 | 3.035E-5 |
| 13 | Development_Glucocorticoid receptor signaling | 5.382E-4 | 0.02756 | 3.151E-5 |
| 14 | Signal transduction_Adenosine A2A receptor signaling pathway | 9.973E-4 | 0.001764 | 3.534E-5 |
| 15 | Immune response_IL-11 signaling pathway via MEK/ERK and PI3K/AKT cascades | 0.01791 | 5.564E-4 | 3.787E-5 |
| 16 | Immune response_Lysophosphatidic acid signaling via NF-kB | 0.007799 | 0.001899 | 4.1E-5 |
| 17 | Inflammatory mechanisms of pancreatic cancerogenesis | 0.003454 | 0.004863 | 4.288E-5 |
| 18 | Immune response_TSLP signalling | 0.02057 | 6.218E-4 | 4.289E-5 |
| 19 | Immune response_PGE2 signaling in immune response | 0.02356 | 7.577E-4 | 6.066E-5 |
| 20 | Growth factors in regulation of oligodendrocyte precursor cells survival in multiple sclerosis | 0.02514 | 8.329E-4 | 7.157E-5 |

### Top 10 Key Gene Hubs

**Early Stage**

| # | Molecular Entity | Molecular Function | Correct Predictions | Activity prediction p-value ▲ |
|---|---|---|---|---|
| 1 | FKBP1B | Binding protein | 2 | 3.133E-4 |
| 2 | miR-20b-5p | RNA | 6 | 4.11E-4 |
| 3 | miR-196b-5p | RNA | 5 | 7.506E-4 |
| 4 | TRPC | Voltage-gated ion-channel | 2 | 9.29E-4 |
| 5 | TUB | Transcription factor | 2 | 9.29E-4 |
| 6 | Bcl-2 | Binding protein | 13 | 1.163E-3 |
| 7 | MVP | Binding protein | 3 | 1.382E-3 |
| 8 | RPL41 | Binding protein | 2 | 1.836E-3 |
| 9 | ORAI3 | Ligand-gated ion-channel | 2 | 1.836E-3 |
| 10 | Protein C receptor (endothelial) | Receptor | 2 | 1.836E-3 |

**Late Stage**

| # | Molecular Entity | Molecular Function | Correct Predictions | Activity prediction p-value ▲ |
|---|---|---|---|---|
| 1 | microRNA 21 | RNA | 9 | 1.376E-5 |
| 2 | c-IAP2 | Binding protein | 8 | 2.735E-5 |
| 3 | ATP1B1 | Binding protein | 5 | 3.603E-5 |
| 4 | miR-210-3p | RNA | 11 | 3.739E-5 |
| 5 | miR-320-3p | RNA | 10 | 5.012E-5 |
| 6 | TNIP1 | Binding protein | 4 | 2.504E-4 |
| 7 | IL-32 (NK4) | Receptor ligand | 4 | 2.504E-4 |
| 8 | TNF-alpha | Receptor ligand | 12 | 2.958E-4 |
| 9 | miR-16-2-3p | RNA | 4 | 3.128E-4 |
| 10 | Pim-2 | Protein kinase | 4 | 3.128E-4 |

**Supplementary Figure 4. Machine Learning Pipeline Outline**. (A) Pre-training configuration of a data matrix (with dimensions samples x features) used for training the classifier involves filtering sample and normalization. (B-C) The CRC classifier is built by training an ensemble of 50 models using 10-fold cross validation. (D) To classify a sample, each model predicts the sample separately and a CRC classifier score is constructed from each model's class prediction based on the proportion of CRC positive calls by the 50 models. If the score is >= 0.5 the sample is classified as CRC+. CRC+: CRC positive call; CRC-: CRC negative call. #CRC+: Number of CRC+ calls made by individual classifier models.

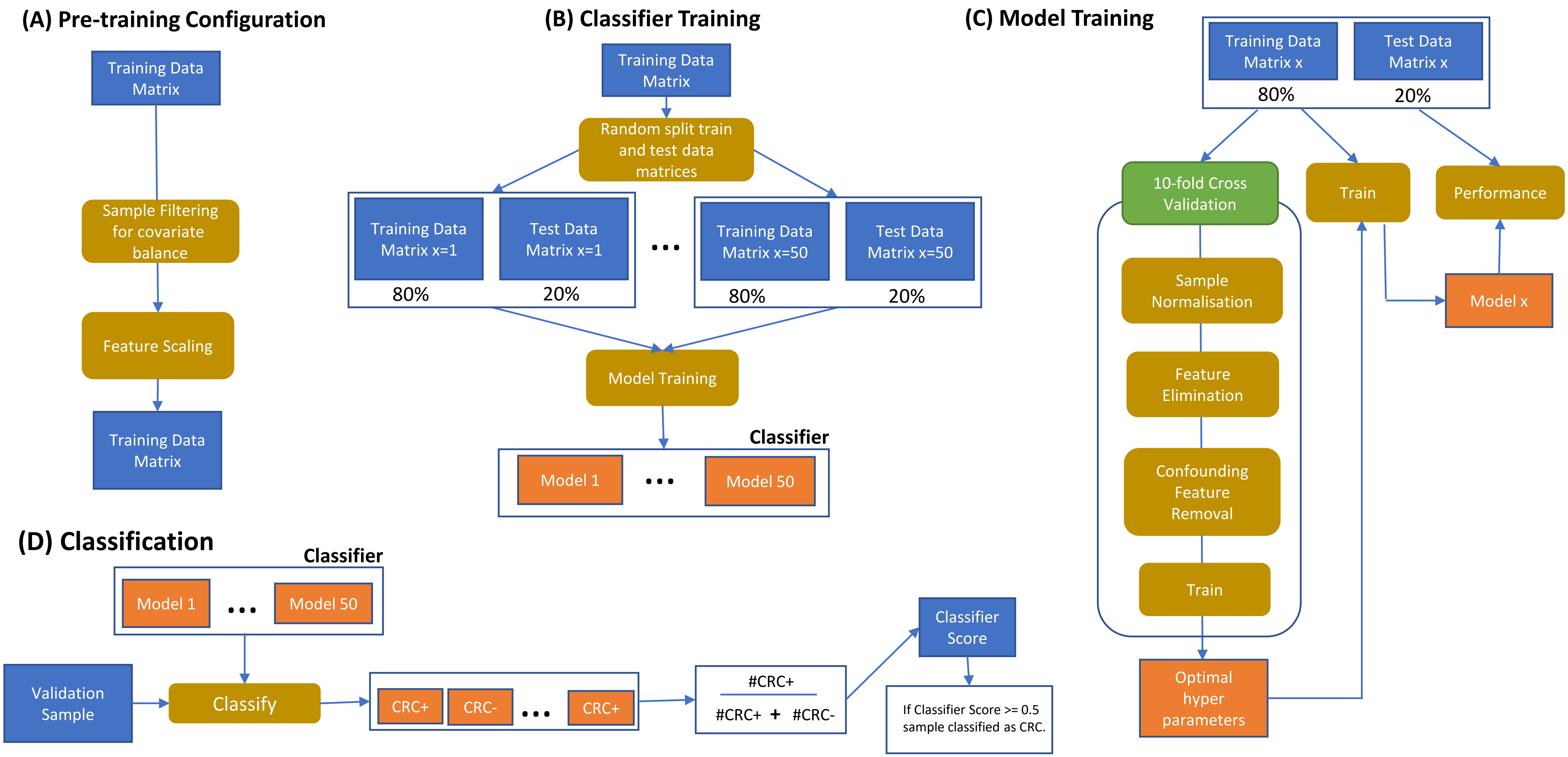

**Supplementary Table 1.** Cut-of-values for classifier sensitivity estimates at 95% specificity on validation samples across each stage

| | % Sensitivity @ 95% Specificity | | | | | Cut-of-values | | | | |
|---|---|---|---|---|---|---|---|---|---|---|
| **Stage** | 1 | 2 | 3 | 4 | All | 1 | 2 | 3 | 4 | All |
| **5hmC** | 55 | 60 | 44 | 66 | 55 | 0.98 | 0.96 | 0.97 | 0.96 | 0.98 |
| **Fragmentomics** | 58 | 51 | 74 | 70 | 62 | 0.84 | 0.83 | 0.84 | 0.84 | 0.83 |
