# Supplementary material for: Hydroxymethylation profile of cell-free DNA is a biomarker for early colorectal cancer
Source: Sci Rep. 2022 Oct 4;12:16566. doi: 10.1038/s41598-022-20975-1 (PMC9532421; doi:10.1038/s41598-022-20975-1)
Supplement: Supplementary file 2 — Supplementary Information 2. [file 41598_2022_20975_MOESM2_ESM.pdf]

Inclusion criteria for the CRC cases was:

a) Subject is diagnosed with colorectal cancer
b) Subject is between 45 and 85 years of age at the time of the blood draw.
c) Subject has had no other cancer
d) If the subject has other GI conditions these should be recorded on the case report form.
e) Consent has been given.

Exclusion criteria for CRC cases was

a) Subject has been diagnosed with any other type of cancers
b) Subject is younger than 45 or older than 85 years old at the time of the blood draw.
c) Subject received neoadjuvant treatment prior to sample collaction .
d) Subject has diagnosis or personal history of any of the following high-risk conditions for colorectal cancer:
   - >= 2 first-degree relatives who have been diagnoses with colon cancer (Note: first-degree relatives include parents, siblings and offspring).
   - One first-degree relative with CRC diagnosed before the age of 60.
   - Familial adenomatous polyposis (also referred to as "FAP", including attenuated FAP)
   - Hereditary non-polyposis colorectal cancer syndrome (also referred to a "HNPCC" or "Lynch Syndrome").
   - Other hereditary cancer syndromes including but are not limited to Peutz-Jeghers Syndrome, MYH-Associated Polyposis (MAP), Gardener's Syndrome, Turcot's (or Crail's) Syndrome, Cowden's Syndrome, Juvenile Polyposis, Cronkhite-Canada Syndrome, Neurofibromatosis and Familial Hyperplastic Polyposis.
e) Subject has family history of
   - Familial adenomatous polyposis (also referred to as "FAP").
   - Hereditary non-polyposis colorectal cancer syndrome (also referred to as "HNPCC" or "Lynch Syndrome").

Inclusion criteria for Adenoma cases

a) Subject is diagnoses with Advanced or Non-advanced colorectal adenoma(s).
b) Subject is between 45 and 85 years of age at the time of the blood draw.
c) Subject has never been diagnosed with any cancer.
d) Consent has been given.

Exclusion criteria for Adenoma cases

a) Subject is younger than 45 or older than 85 years old at the time of the blood draw.
b) Subject has a history of colorectal cancer.
c) Subject has been diagnosed or has a history of any cancer.
d) Subject had prior colorectal resection for any reason other than sigmoid diverticular disease.
e) Subject had overt rectal bleeding, e.g. hematochezia or melena, within the previous 30 days.
f) Subject has diagnosis or personal history of any of the following high-risk conditions for colorectal cancer:
   - >= 2 first-degree relatives who have been diagnoses with colon cancer (Note: first-degree relatives include parents, siblings and offspring).
   - One first-degree relative with CRC diagnosed before the age of 60.

- Familial adenomatous polyposis (also referred to as “FAP”, including attenuated FAP)
- Hereditary non-polyposis colorectal cancer syndrome (also referred to a “HNPCC” or “Lynch Syndrome”).
- Other hereditary cancer syndromes including but are not limited to Peutz-Jeghers Syndrome, MYH-Associated Polyposis (MAP), Gardener’s Syndrome, Turcot’s (or Crail’s) Syndrome, Cowden’s Syndrome, Juvenile Polyposis, Cronkhite-Canada Syndrome, Neurofibromatosis and Familial Hyperplastic Polyposis.

g) Subject has family history of
- Familial adenomatous polyposis (also referred to as “FAP”).
- Hereditary non-polyposis colorectal cancer syndrome (also referred to as “HNPCC” or “Lynch Syndrome”).

Inclusion criteria of the Controls

a) Subject has average risk for development of colorectal cancer (as defined by the exclusion criteria below).
b) Subject is between 45 and 85 years of age at the time of the blood draw.
c) Subject has no history of colorectal cancer and has no adenomas, as determined by colonoscopy.
d) If the subject has attended colonoscopy for any other reason other than routine screening only then this must be recorded on the case report form.
e) If the subject has other GI conditions these should be recoded on the case report form.
f) Consent has been given.

<u>Exclusion criteria for Controls</u>

a) Subject is younger than 45 or older than 85 years old at the time of the blood draw.
b) Subject has a history of colorectal cancer or adenoma as determined by colonoscopy.
c) Subject has been diagnosed or has a history of any cancer.
d) Subject had a positive fecal occult blood test or FIT within the previous 12 months.
e) Subject had prior colorectal resection for any reason other than sigmoid diverticular disease.
f) Subject had overt rectal bleeding, e.g. hematochezia or melena, within the previous 30 days.
g) Subject has diagnosis or personal history of any of the following high-risk conditions for colorectal cancer:
   - >= 2 first-degree relatives who have been diagnoses with colon cancer (Note: first-degree relatives include parents, siblings and offspring).
   - One first-degree relative with CRC diagnosed before the age of 60.
   - Familial adenomatous polyposis (also referred to as “FAP”, including attenuated FAP)
   - Hereditary non-polyposis colorectal cancer syndrome (also referred to a “HNPCC” or “Lynch Syndrome”).
   - Other hereditary cancer syndromes including but are not limited to Peutz-Jeghers Syndrome, MYH-Associated Polyposis (MAP), Gardener’s Syndrome, Turcot’s (or Crail’s) Syndrome, Cowden’s Syndrome, Juvenile Polyposis, Cronkhite-Canada Syndrome, Neurofibromatosis and Familial Hyperplastic Polyposis.
h) Subject has family history of

- Familial adenomatous polyposis (also referred to as “FAP”).
- Hereditary non-polyposis colorectal cancer syndrome (also referred to as “HNPCC” or “Lynch Syndrome”).

i) Subject is having a colonoscopy to diagnose unexplained rectal bleeding or intestinal blockage, or other symptoms suggestive of colorectal cancer. Subjects that are receiving colonoscopies to aid diagnosis of IBD or other, non-malignat, diseases should not be excluded.

j) No more than 10% of controls can have received a diagnosis of inflammatory bowel disease.
